# Classical Swine Fever Virus p7 Protein Interacts with Host Protein CAMLG and Regulates Calcium Permeability at the Endoplasmic Reticulum

**DOI:** 10.3390/v10090460

**Published:** 2018-08-28

**Authors:** Douglas P. Gladue, Eneko Largo, Lauren G. Holinka, Elizabeth Ramirez-Medina, Elizabeth A. Vuono, Keith A. Berggren, Guillermo R. Risatti, Jose L. Nieva, Manuel V. Borca

**Affiliations:** 1 Plum Island Animal Disease Center, ARS, USDA, Greenport, NY 11944, USA; Douglas.Gladue@ars.usda.gov (D.P.G.); Lauren.Holinka-patterson@jax.org (L.G.H.); Elizabeth.Ramirez@ars.usda.gov (E.R.-M.); Elizabeth.Vuono2@ars.usda.gov (E.A.V.); keithab@princeton.edu (K.A.B.); 2 Biofisika Institute, University of the Basque Country (CSIC-UPV/EHU), 48940 Leioa, Spain; eneko.largo@gmail.com (E.L.); joseluis.nieva@ehu.eus (J.L.N.); 3 Department of Pathobiology and Veterinary Science, University of Connecticut, Storrs, CT 06269, USA; Guillermo.Risatti@uconn.edu; 4 Oak Ridge Institute for Science and Education (ORISE), Oak Ridge, TN 37830, USA

**Keywords:** viroporin, CSFV, CSF, CAMLG, classical swine fever

## Abstract

We have previously shown that Classical Swine Fever Virus (CSFV) p7 is an essential nonstructural protein with a viroporin activity, a critical function in the progression of virus infection. We also identified p7 domains and amino acid residues critical for pore formation. Here, we describe how p7 specifically interacts with host protein CAMLG, an integral ER transmembrane protein involved in intracellular calcium release regulation and signal response generation. Detection of interaction as well as the identification of p7 areas mediating interaction with CAMLG was performed by yeast two-hybrid. p7-CAMLG interaction was further confirmed by confocal microscopy in eukaryotic cells, co-expressing both proteins. Mutant forms of p7 having substituted native residues identified as mediating interaction with CAMLG showed a decreased co-localization compared with the native forms of p7. Furthermore, it is shown that native p7, but not the mutated forms of p7 that fail to interact with CAMLG, efficiently mediates calcium permeability in the ER. Interestingly, viruses harboring some of those mutated forms of p7 have been previously shown to have a significantly decreased virulence in swine.

## 1. Introduction

Classical swine fever virus (CSFV) is the causative agent of a highly contagious economically significant viral disease of domestic and wild pigs. CSFV is a member of the genus *Pestivirus* in the family *Flaviviridae* along with two other viruses of significant veterinary importance—bovine viral diarrhea virus (BVDV) and border disease virus (BDV) [1]. The CSFV genome, approximately 12 kb length, comprises of only one open reading frame encoding a unique polyprotein that, after posttranslational processing, originates 12 individual proteins: NH2-Npro-C-Erns-E1-E2-p7-NS2-NS3-NS4A-NS4BNS5A-NS5B-COOH [2,3].

The nonstructural protein p7 is an essential small hydrophobic transmembrane protein of approximately 6–7 kDa. Structurally, p7 possesses a short area of charged residues that is flanked by stretches of hydrophobic amino acids, which are predicted to constitute a cytosolic loop and two transmembrane helices, respectively [4]. Our laboratory has shown that p7 is a class II viroporin. We demonstrated, using an in vitro model emulating the Endoplasmic Reticulum (ER) membrane composition, that the C-terminal transmembrane helix of p7 possesses pH-dependent pore forming activity [4]. Furthermore, we also demonstrated that pore formation in our ER modeled membranes by the p7 C-terminal domain depends on two sequence determinants of the protein: the C-terminal transmembrane helix (comprised by residues 39–67), and the preceding polar loop (residues 33–38), which regulates the pore forming activity [5]. In addition, we showed that p7 actually induces two types of pores with slightly different sizes and opposite ion selectivity [6]. Specific amino acid substitutions affecting conserved residues within these areas of the protein severely affect ER-like membrane permeabilization [7].

To better characterize the possible role of p7 during virus infection, we have identified host cell proteins that interact with p7 using a yeast two-hybrid approach. Interestingly, we found that p7 interacts with CAMLG, an integral ER transmembrane protein [8]. CAMLG was originally found to interact with cyclophilin B, facilitating the calcium-dependent activation of nuclear factor of the activated T-cells [9]. Structurally, the C-terminus of CAMLG is integrated into the membrane, but most of molecule faces the cytoplasm [8]. The CAMLG cytoplasmic tail interacts with the intracellular portions of several membrane-associated receptors such as those for epidermal growth factor, mucin, and GABA [10,11,12]. In fact, CAMLG expression elevates the cytosolic calcium concentration, activating cellular transcription activity involving increased intracellular calcium mobilization [13,14].

We also mapped areas of p7 mediating interaction with CAMLG, as determined by yeast two-hybrid. Interaction between p7 and CALMG was further confirmed in eukaryotic cells expressing both proteins by confocal microscopy. Mutant forms of p7 having substituted native residues mediating p7-CAMLG interaction presented a decreased co-localization compared with the native forms of p7. Native p7, but not the mutated forms failing to interact with CAMLG, efficiently mediates calcium permeability in the ER. Interestingly, viruses harboring some of those mutated forms of p7 have been previously shown by us to have a significantly decreased virulence in swine.

## 2. Materials and Methods

### 2.1. Cells and Viruses

CSFV stocks were prepared in swine kidney cells (SK6) [15]. SK6 cells, free of BVDV, were cultured in Dulbecco’s Minimal Essential Media (DMEM) (Gibco, Grand Island, NY, USA) using 10% fetal calf serum (FCS) (Atlas Biologicals, Fort Collins, CO, USA). CSFV Brescia strain was derived from our CSFV Brecia infectious cDNA clone (IC) [16]. Virus growth kinetics assessment was simultaneously performed on SK6 cells [17] and primary swine macrophage cell cultures, which were prepared as previously described [18]. In both cases, cell monolayers were prepared in 24-well plates and infected at a MOI of 0.01 (based on TCID_50_ previously determined in SK6 cell cultures). Cell cultures were kept at 37 °C under 5% CO_2_. After 1 h of adsorption, the inoculum was removed, cell monolayers rinsed three times with PBS, once with media and then incubated for 2, 24, 48, 72 and 96 h at 37 °C under 5% CO_2_. At appropriate times post-infection (pi), the cells were frozen at ≤−70 °C and the thawed lysates were used to determine titers. All samples were titrated simultaneously, to avoid inter-assay variability, using SK6 cells in 96-well plates (Costar, Cambridge, MA, USA). Presence of infectious virus was detected, by the 4th day post infection (pi), by an immunoperoxidase assay using the CSFV monoclonal antibody WH303 [16] and the Vectastain ABC kit (Vector Laboratories, Burlingame, CA, USA). Titers were calculated using the method of Reed and Muench [19] and expressed as TCID_50_/mL. As performed, test sensitivity was ≥1.8 TCID_50_/mL.

Methodology to develop CSFV harboring mutated forms of p7 was performed by introducing alanine substitutions into a full-length IC of the virulent CSFV Brescia strain (pBIC) as previously described [4].

Human embryonic kidney cells, 293T were used in ER localization studies and are available from (American Type Culture Collection, Manassas, VA, USA) catalog number: CRL-3216.

### 2.2. Development of the cDNA Library

A porcine macrophage yeast two-hybrid library was constructed (Clontech, Mountain View, CA, USA) using cDNA obtained from swine monocytes/macrophages. Macrophage cultures were prepared as previously described [20]. The Gal4-AD expression vector was used to express cellular proteins obtained from macrophage cDNA.

### 2.3. Library Screening

The yeast two-hybrid GAL4 system detects protein-protein interactions as previously described [21,22]. In this study, CSFV p7 protein was expressed as a fusion protein to the GAL4 Binding Domain (BD). Full-length p7 protein (amino acid residues 1062–1129 of the CSFV strain Brescia polyprotein) was used for screening and mutant studies. Screening was conducted with the cDNA swine macrophage library by using histidine and adenine reporter genes to select positive interacting host proteins using growth selection. Selection was done on media that also lacked tryptophan and leucine to allow for plasmid maintenance. More details on the screening process have been previously described [23]. In this study, we used as a negative control protein Lam which is Human Lamin C, as it is well established that Lamin C is not often involved in protein complexes. The CAMLG clone recovered from the library was identified as porcine CAMLG (NCBI Reference Sequence: NP_001231822.1) and was amino terminal fused in frame with the GAL4 activation domain.

**Figure 1:**
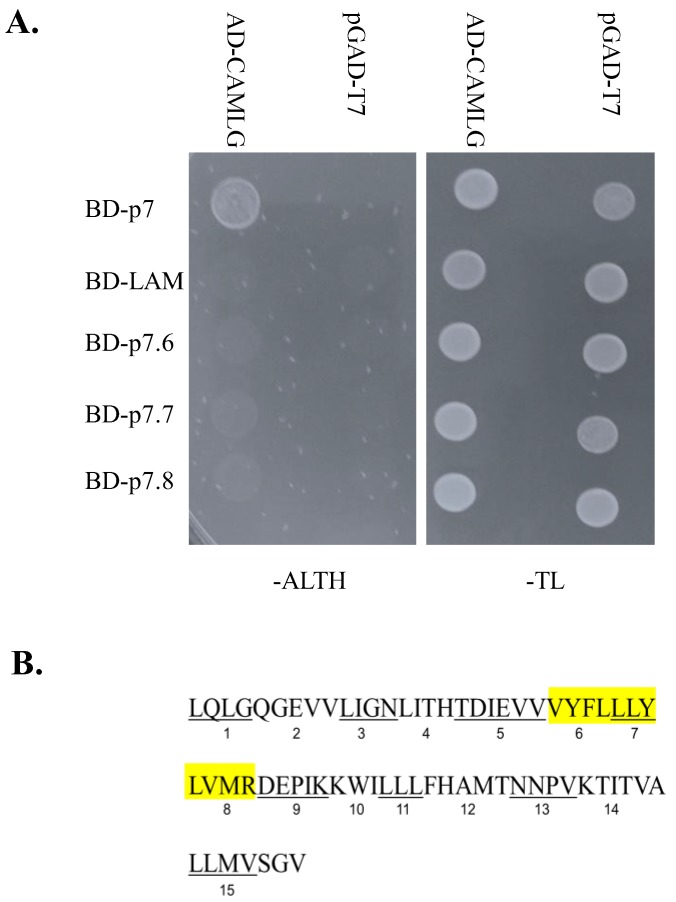
Interaction of CSFV p7 protein and host CAMLG detected by yeast two-hybrid system. (**A**) Yeast strain AH109 was transformed with either GAL4-binding domain (BD) fused to CSFV p7 (BD-p7), or mutated CSFV p7 construct as indicated or human lamin C (BD-LAM), a negative control and GAL4 activation domain (AD) fused to CAMLG (AD-CAMLG) or the negative control pGADT7 as indicated. 2 × 10^6^ yeast cells were spotted onto either SD-Ade/His/Leu/Trp plates (-ALTH), selective media for detecting protein-protein interactions or nonselective SD-Leu/Trp plates (-TL) (**B**) Schematic representation of CSFV p7 alanine mutants used in the present study. The name of each alanine p7 mutant is shown below the mutated amino acid residues for each particular mutant. All indicated residues were mutated to an alanine. The highlighted p7 mutants indicate alanine substitutions resulting in lack of binding of p7 to CAMLG in the yeast two-hybrid system.

Mapping CAMLG interacting area in p7 was performed by using a library of mutated forms of p7 where specific areas of native p7 encoding for groups of three to six amino acid residues were substituted with alanine codons (Figure 1B). Alanine substitutions of p7 native residues were introduced by site-directed mutagenesis (using the QuikChange XL site-directed mutagenesis kit) using the full-length pBIC as a DNA template as previously described [24]. Primers were designed using the Stratagene primer mutagenesis program.

### 2.4. p7-CAMLG Co-Localization Studies

For these studies, p7 was tagged on its C-terminus with GFP (p7-GFP) while CAMLG was tagged in its N-terminus with RFP (RFP-CAMLG). In both cases, reporter genes were place under the control of the CMV promoter. p7-GFP and RFP-CAMLG plasmids were synthetized by Epoch Life Sciences. Sub-confluent monolayers of SK6 cells were grown in 6-well plates and co-transfected with Fugene HD (Active Motif, Carlsbad, CA, USA) as described by the manufacturer. After 24 hours, the cells were split on 12 mm glass cover-slips, and fixed with 4% paraformaldehyde (EMS, Hatfield, PA, USA) for 15 minutes. Paraformaldehyde was then removed, the cover-slips washed three times with PBS and then they were mounted with prolong gold containing DAPI (Life Technologies, Carlsbad, CA, USA) and left for 24 h to cure. Preparations were examined using a Zeiss LSM710 fluorescence microscope (Oberkochen, Germany) and captured images adjusted for contrast and brightness using ZEN black 2012 software (Oberkochen, Germany).

### 2.5. ER Localization and Permeabilization to Calcium

ER localization of the expressed p7-GFP fusion protein, and its derived mutants, was assayed by co-transfecting 293T cells (2 × 10^5^ cells) with plasmids encoding p7-GFP fusions and mCh-Sec61 beta (1 µg each) using calcium phosphate as described elsewhere [25]. Under these conditions, 70–80% of the cells in the culture were successfully transfected. Thirty-six hours post-transfection, cells were fixed with 4% formaldehyde in PBS and incubated with Hoechst dye. Confocal images were obtained on a Leica TCS SP5 II microscope (Leica Microsystems GmbH, Wetzlar, Germany), using an 63× water-immersion objective.

Levels of ER permeabilization were determined through fluorescence microscopy of transfected single cells by following the amount of thapsigargin-releasable Ca^2+^ in the cytosol [26,27,28]). To that end, changes in fluorescence intensity of the indicator probe rhod-2/AM were recorded as a function of time [28,29,30]. At 36 h after transfection, cells were loaded with rhod-2/AM (4 µg/mL) at room temperature for 45 min. Dye-loaded cells were visualized in confocal mode in a Nikon Eclipse TE-2000 inverted microscope, after excitation with a 561-nm (Yellow-Green) laser and emission collected with dichroic mirrors set at 593/40 nm. Time-lapse images were recorded on a high sensitivity Nikon D-Eclipse C1 Si color camera at a frame acquisition interval of 1 s.

## 3. Results

### 3.1. Cloning of the p7 Gene and Screening the Swine-Macrophage cDNA Library

CSFV p7 gene was amplified by PCR using the following forward and reverse primers: 5′ GCTAGGAATTCCTACAGTTAGGCCAGGGTGAG 3′ and 5′ GTCGACTTAAACCCCGCTA ACCATGAGCA 3′, containing flanking EcoRI and Sal1 sites, respectively. The resulting product was digested with EcoRI and Sal1, and cloned into the corresponding restriction enzyme sites into the pGBKT7 vector. Resulting clones were sequenced to verify fidelity of the p7 sequence and that they were in frame with the GAL4 DNA binding domain (BD). The resulting plasmid pBD-p7 was transformed into yeast strain AH109, along with a negative control -pGADT7, the empty library vector containing the GAL4 Activation Domain (AD) to confirm that there was no self-activation of pBD-p7. (Figure 1A).

The custom-made swine macrophage cDNA library fused to the AD contains >3 × 10^6^ individual cDNA clones; therefore, our screen of 1 × 10^7^ colonies represents a greater than 3-fold library saturation. Initially, we identified 17 individual open reading frames (ORFs); however, after sequence analysis, there was only one protein identified, calcium modulating ligand (CAMLG).

To assess the specificity of the interaction between the CSFV p7 and host protein CAMLG, the yeast strain AH190 was co-transformed with pGADT7 or pAD-CAMLG and pBD-p7 or proteins as negative controls BD-Lam (a plasmid containing human Lamin C). The resulting colonies that grew at 30 °C on solid plasmid selective media lacking tryptophan and leucine (SD-trp/leu) were then grown overnight in liquid media and 1 × 10^6^ cells were spotted on SD-trp/leu and media also lacking adenine and histidine (SD-trp/leu/ade/his). Positive protein-protein interactions are expected to activate transcription of both the adenine and histidine reporter genes, allowing for growth on media lacking adenine and histidine. AD-CAMLG were shown to specifically interact with BD-p7 and not with the negative controls (Figure 1A).

### 3.2. Mapping of Interaction Domain in p7 with CAMLG

To identify the amino acid residues of p7 mediating the interaction with CAMLG, an alanine scanning mutagenesis was performed on the CSFV p7 gene. We successfully used this particular approach for mapping specific areas within FMDV viral proteins specifically recognized by host proteins [31,32]. As illustrated in Figure 1B, contiguous stretches (containing 3 to 7 residues) of p7 native amino acid residues were substituted with alanine through site-directed mutagenesis. As a result, 13 mutated constructs were made encompassing the entire length of p7. Then, each alanine-mutated form of p7 was tested individually for binding with proteins pAD-CAMLG or pGADT7, as a negative control. The resulting transformants were grown on SD-trp/leu/ade/his and as a control SD-trp/leu. Alanine-mutated areas of p7 that lose binding with CAMLG protein are considered those actively involved in the interaction with the host protein. The areas of p7 losing reactivity with CAMLG were p7.6 (comprising p7 residues 25–28), p7.7 (comprising p7 residues 29–31), and p7.8 (comprising p7 residues 32–35), indicating that the area of p7 between amino acid residues 25 and 35 is involved in mediating binding with CAMLG (Figure 1B).

### 3.3. Interaction of p7 with CAMLG in Eukaryotic Cells

**Figure 2:**
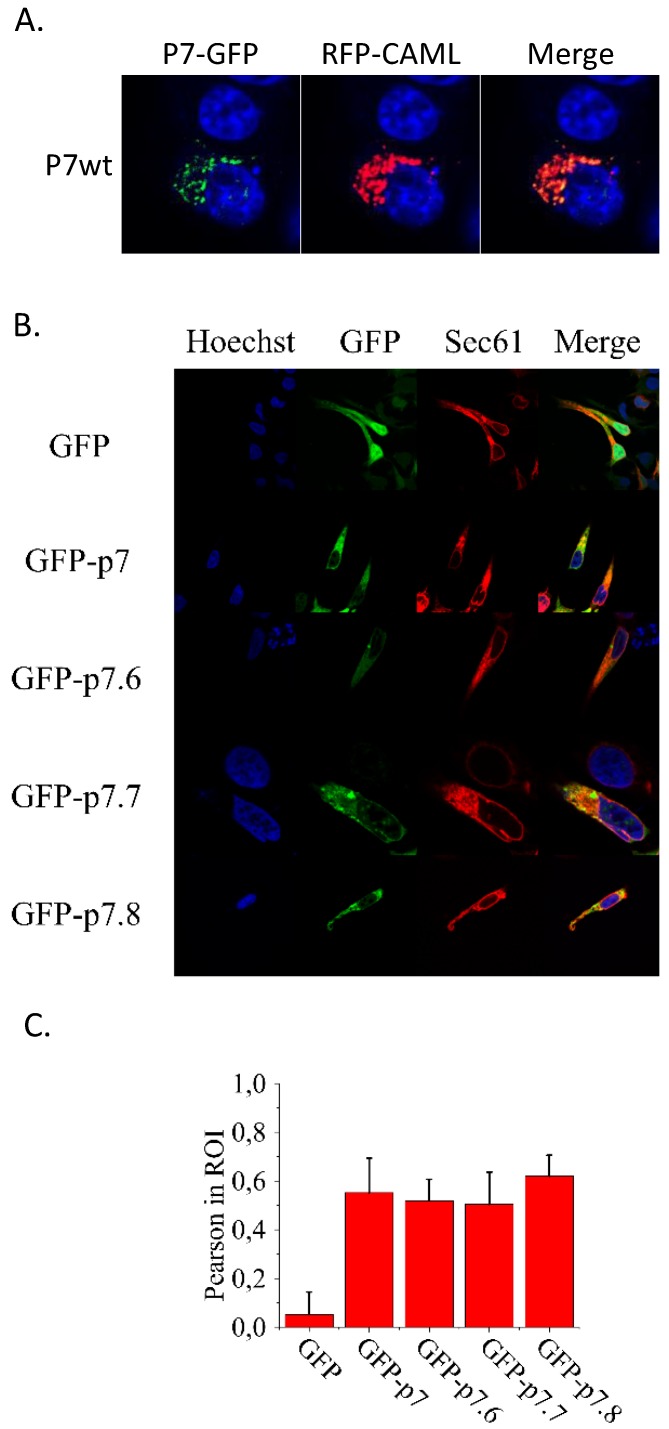
(**A**) Co-localization of p7 and CAMLG in transfected SK6 cells imaged at 1000×. p7-GFP and RFP-CAMLG constructs were co-transfected in SK6 cell monolayers. p7 mutant constructs fused to GFP (7.6-GFP, 7.7-GFP and 7.8-GFP) were co-transfected with RFP-CAMLG in SK6 cells. Preparations were mounted with ProLong Gold antifade reagent containing DAPI to stain the nuclei. In the merged panel, yellow color indicates co-localization of GFP and RFP. (**B**) Transfection Native and mutated forms of p7 in 293T cells imaged at 630× located in the endoplasmic reticulum. Distribution of p7 and its mutants 7.6, 7.7 and 7.8 by confocal microscopy. Micrographs illustrate individual cells co-expressing GFP constructs and the ER marker mCh-Sec61. The control GFP devoid of membrane anchors labelled the complete cell (top panels), whereas GFP-p7 constructs were excluded from the nucleus and co-localized with mCh-Sec61. (**C**) Co-localization of mCh-Sec6 and GFP in the samples as calculated with the ImageJ plugin Coloc 2 program (http://imagej.net/Coloc_2). Measurements were carried out in at least 6 cells as those displayed in the previous panel. Bars represent mean values ± SE.

To confirm that interaction between p7 and CAMLG proteins happens in swine cells, two plasmids were prepared: one containing p7 tagged on its C-terminus with GFP (p7-GFP) and a second where CAMLG was tagged in its N-terminus with RFP (RFP-CAMLG). Swine kidney cells (SK6) cultures were co-transfected with both plasmids and observed for co-localization. Results demonstrate that cells that express both plasmids present co-localization of p7-GFP and RFP-CAMLG, making numerous circular overlapping structures throughout the cell, confirming that p7 and CAMLG co-localize in a swine cell line (Figure 2A).

### 3.4. Location of Native and Mutated Forms of p7 in the Endoplasmic Reticulum and Their Effects on Calcium Release

Before determining the effects of the studied mutations in calcium release from the ER, co-localization analyses were carried out to confirm that they did not affect the biogenesis and assembly of p7 within this organelle. Thus, confocal microscopy of transfected 293T cells revealed similar co-localization with the ER marker mCh-Sec6 for the native GFP-p7 and its mutated forms GFP-p7.6, GFP-p7.7 and GFP-p7.8 (Figure 2B). This finding was further demonstrated by the comparable Pearson indexes calculated for co-localization with mCh-Sec6 in all cases (Figure 2C). Therefore, substitution of native amino acid sequence by alanine residues in mutants p7.6, p7.7 and p7.8 does not alter per se their expression or cell localization when compared with the parental p7.

**Figure 3:**
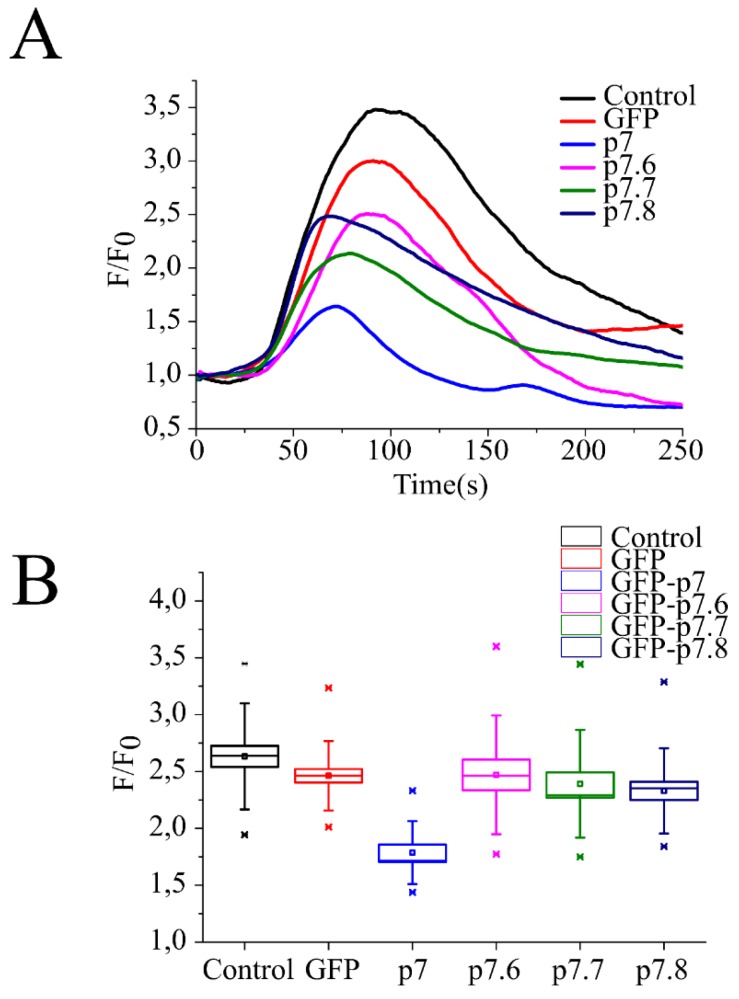
Effect of native and mutated forms of p7 in thapsigargin-induced calcium release from the endoplasmic reticulum. (**A**) Kinetic traces of Ca^2+^ efflux from the ER into the cytosol in cells transfected with GFP or the GFP fusions as indicated in the panel. (**B**) Maximal extents of rhod-2/AM intensity change upon thapsigargin addition.

Experiments quantitatively imaging the cytosolic calcium levels with the Ca^2+^ indicator rhod-2/AM were next conducted to compare the effects of the native and mutated forms of p7 (Figure 3). Thapsigargin blocks the Ca^2+^-ATPase of internal stores [28]. Addition of this compound to control (non-transfected) and GFP-expressing 293T cells caused comparable increases in rhod-2/AM fluorescence, following the rise in the cytosolic concentration of calcium (Figure 3A). Rhod-2/AM fluorescence changes of comparable extent were recorded in cells expressing the GFP-p7.6, GFP-p7.7 or GFP-p7.8 proteins, suggesting that the ER containing these proteins retained the same concentration of thapsigargin-releasable calcium than the untreated controls (Figure 3B). In contrast, in cells expressing the parental sequence GFP-p7, thapsigargin addition resulted in rhod-2/AM fluorescence changes that were significantly reduced, consistent with the permeabilization state of the organelle with respect to calcium. Therefore, alanine substitutions of the native amino acid residues in mutants p7.6, p7.7 and p7.8 profoundly alter the ability of parental p7 protein to mediate ER permeabilization to Ca^2+^.

### 3.5. Growth Ability of Recombinant CSFV Harboring Mutations in p7

**Figure 4:**
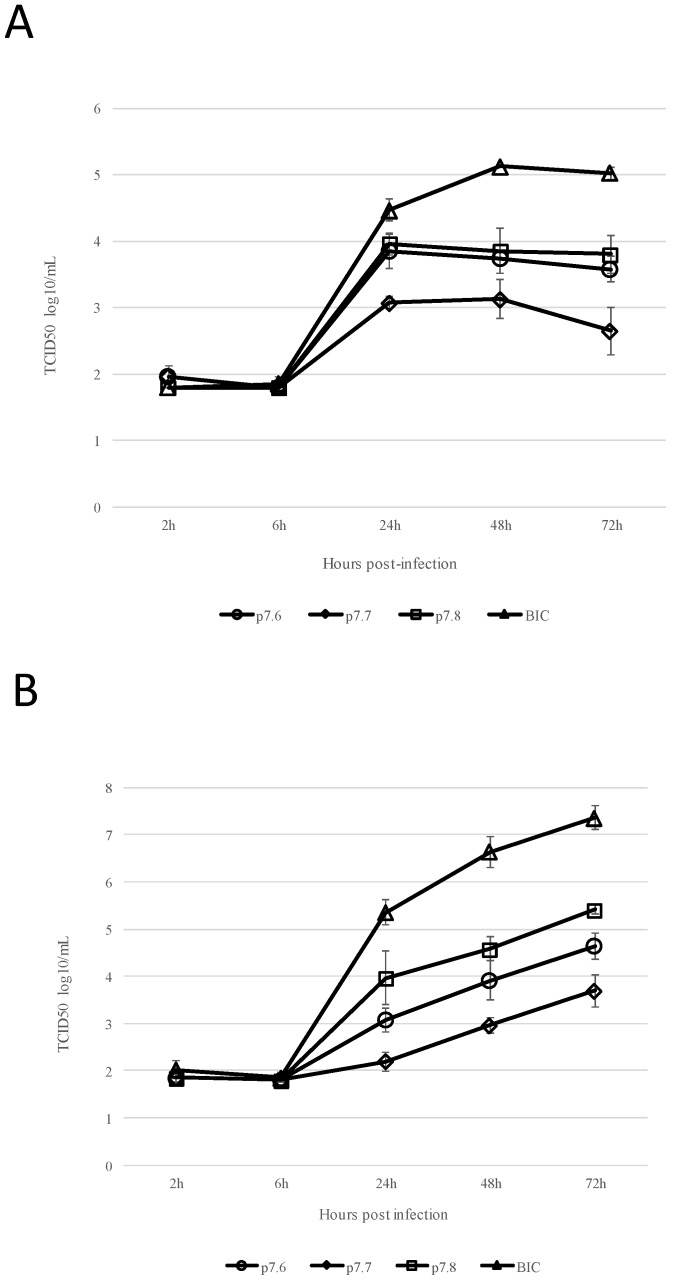
In vitro growth kinetics of CSFV mutants 7.6, 7.7 and 7.8 and parental CSFV BICv. (**A**) SK6 or (**B**) swine macrophage cell cultures were infected (MOI0.01). Virus yields, were titrated in SK6 cell cultures as described in Material and Methods. Data represent means and standard deviations from two independent experiments. Sensitivity of virus detection: ≥log10 1.8 HAD50/mL.

We previously reported the production of recombinant CSFV harboring p7 mutated forms 7.6, 7.7 and 7.8 [4]. In that report, we showed that introduction of p7.6, p7.7 and p7.8 in an infectious clone encoding for highly virulent isolate Brescia were compatible with virus replication. In order to assess the effect of mutated forms of p7 (7.6, 7.7 and 7.8) in virus growth kinetics, replication of these recombinant CSFVs was evaluated in vitro in a multistep growth curve using as substrate either SK6 cell line or primary swine macrophage cell cultures (the cell targeted by CSFV during natural infection in swine) and compared to parental BICv. Cell cultures were infected with the corresponding virus (at a MOI of 0.01) and samples were collected at 2, 6, 24, 48, 72 and 96 h pi. Samples were titrated in SK6 cells. In both cell substrates, all three mutant viruses showed a significant decreased ability to replicate. In both cell substrates, this disability was more pronounced in mutant p7.7, intermediate in p7.6, and less evident in p.7.8, although titers at 72 h pi were at least 10-fold lower for p7.8 mutant than for parental BICv (Figure 4A,B). Therefore, mutations in p7 altering CAMLG binding severely affect the ability of CSFV to replicate in cell cultures.

## 4. Discussion

CSFV nonstructural protein p7 has been previously shown to be a virally encoded pore forming protein, or viroporin, which is essential for virus replication as well as for virulence in swine [4]. p7 has also been further characterized in terms of the protein domains and critical residues involved in pore forming activities [5,6,7]. However, little is known about other functions of p7 or the possible interaction of p7 with the host proteins during virus replication.

The central purpose of this work was the identification of host proteins that interact with CSFV viral nonstructural protein p7, and the potential role of that interaction in the process of virus replication and/or virus virulence. Host protein CAMLG was found to specifically interact with p7 in the yeast two-hybrid. Interestingly, CAMLG has been reported to be involved with the modulation cellular pore function. Thus, expression of CAMLG increases the concentration of cytosolic calcium leading to the stimulation of the cellular NF-AT transcription activity [14]. It has been also shown that CAMLG is involved in the intracellular calcium increased mobilization, which activates signal transduction and activation required for the prolactine-dependent growth of breast cancer cells [13]. It has been well established that variations in the concentration of cytosolic calcium trigger critical cellular functions and modulates cell metabolism [33]. Besides, calcium concentration also has a key function in generating mitotic division in several cell types and, on the contrary, in the regulation of cell death [34]. It is well accepted that severe calcium dysregulation promotes cell death through necrosis and a more controlled intracellular increases in calcium concentration induced by milder stimulus may promote cell death through apoptosis [33].

Therefore, it could be speculated that p7 directly or through its interaction with CAMLG may be important in cell signaling or activation during the CSFV replication cycle. In fact, we demonstrate here that p7 efficiently increases intracellular Ca^2+^. It is not clear if this phenomenon is directly mediated by p7 or results from its interaction with CAMLG. Interestingly, disruption of that interaction by introducing mutations in mutants p7.6, p7.7 and p7.8 decreased the ability of p7 to affect increased release of intracellular Ca^2+^.

In addition, CAMLG has been demonstrated to be involved in modulation of apoptosis during viral infections. CAMLG interacts with Dengue virus protein C and with protein pK7 of Kaposi’s sarcoma-associated herpesvirus (KSHV) preventing or modulating cell apoptosis, respectively [35,36]. It is possible that during CSFV infection that p7 could modulate CAMLG in a similar manner as KSHV or Dengue to prevent apoptosis in CSFV-infected cells, as CSFV-infected cells show no evidence of apoptosis [1]; however, more work will be needed to determine if this is the case.

Results presented here constitute the first study discovering host proteins that interact with CSFV nonstructural protein p7. In addition, the first demonstration that p7 by itself has the ability to modulate intracellular calcium concentrations. By developing a better understanding of host proteins directly interacting with CSFV protein p7, we may gain a more comprehensive understanding of the role of p7 protein during virus replication and infection. Further work will be necessary to evaluate if this information may help in the development of specific antivirals or virus counter measures.

## References

[1] Becher P., Avalos Ramirez R., Orlich M., Cedillo Rosales S., Konig M., Schweizer M., Stalder H., Schirrmeier H., Thiel H.J. (2003). Genetic and antigenic characterization of novel pestivirus genotypes: Implications for classification. Virology.

[2] Lamp B., Riedel C., Roman-Sosa G., Heimann M., Jacobi S., Becher P., Thiel H.J., Rumenapf T. (2011). Biosynthesis of classical swine fever virus nonstructural proteins. J. Virol..

[3] Thiel H.J., Stark R., Weiland E., Rumenapf T., Meyers G. (1991). Hog cholera virus: Molecular composition of virions from a pestivirus. J. Virol..

[4] Gladue D.P., Holinka L.G., Largo E., Fernandez Sainz I., Carrillo C., O’Donnell V., Baker-Branstetter R., Lu Z., Ambroggio X., Risatti G.R. (2012). Classical swine fever virus p7 protein is a viroporin involved in virulence in swine. J. Virol..

[5] Largo E., Gladue D.P., Huarte N., Borca M.V., Nieva J.L. (2014). Pore-forming activity of pestivirus p7 in a minimal model system supports genus-specific viroporin function. Antivir. Res..

[6] Largo E., Verdia-Baguena C., Aguilella V.M., Nieva J.L., Alcaraz A. (2016). Ion channel activity of the CSFV p7 viroporin in surrogates of the ER lipid bilayer. Biochim. Biophys. Acta.

[7] Largo E., Gladue D.P., Torralba J., Aguilella V.M., Alcaraz A., Borca M.V., Nieva J.L. (2018). Mutation-induced changes of transmembrane pore size revealed by combined ion-channel conductance and single vesicle permeabilization analyses. Biochim. Biophys. Acta.

[8] Holloway M.P., Bram R.J. (1998). Co-localization of calcium-modulating cyclophilin ligand with intracellular calcium pools. J. Boil. Chem..

[9] Bram R.J., Crabtree G.R. (1994). Calcium signalling in T cells stimulated by a cyclophilin B-binding protein. Nature.

[10] Guang W., Twaddell W.S., Lillehoj E.P. (2012). Molecular Interactions between MUC1 Epithelial Mucin, beta-Catenin, and CagA Proteins. Front. Immunol.

[11] Tran D.D., Russell H.R., Sutor S.L., van Deursen J., Bram R.J. (2003). CAML is required for efficient EGF receptor recycling. Dev. Cell.

[12] Yuan X., Yao J., Norris D., Tran D.D., Bram R.J., Chen G., Luscher B. (2008). Calcium-modulating cyclophilin ligand regulates membrane trafficking of postsynaptic GABA(A) receptors. Mol. Cell Neurosci..

[13] Lim J.H., Kim T.Y., Kim W.H., Park J.W. (2011). CAML promotes prolactin-dependent proliferation of breast cancer cells by facilitating prolactin receptor signaling pathways. Breast Cancer Res. Treat..

[14] Von Bulow G.U., Bram R.J. (1997). NF-AT activation induced by a CAML-interacting member of the tumor necrosis factor receptor superfamily. Science.

[15] Terpstra C., Woortmeyer R., Barteling S.J. (1990). Development and properties of a cell culture produced vaccine for hog cholera based on the Chinese strain. DTW. Dtsch. Tierarztl. Wochenschr..

[16] Risatti G.R., Borca M.V., Kutish G.F., Lu Z., Holinka L.G., French R.A., Tulman E.R., Rock D.L. (2005). The E2 glycoprotein of classical swine fever virus is a virulence determinant in swine. J. Virol..

[17] Risatti G.R., Holinka L.G., Lu Z., Kutish G.F., Tulman E.R., French R.A., Sur J.H., Rock D.L., Borca M.V. (2005). Mutation of E1 glycoprotein of classical swine fever virus affects viral virulence in swine. Virology.

[18] Zsak L., Lu Z., Kutish G.F., Neilan J.G., Rock D.L. (1996). An African swine fever virus virulence-associated gene NL-S with similarity to the herpes simplex virus ICP34.5 gene. J. Virol..

[19] Reed L.J., Muench H. (1938). A simple method of estimating fifty percent endpoints. Am. J. Hyg..

[20] Gladue D.P., Holinka L.G., Fernandez-Sainz I.J., Prarat M.V., O’Donell V., Vepkhvadze N., Lu Z., Rogers K., Risatti G.R., Borca M.V. (2010). Effects of the interactions of classical swine fever virus Core protein with proteins of the SUMOylation pathway on virulence in swine. Virology.

[21] Chien C.T., Bartel P.L., Sternglanz R., Fields S. (1991). The two-hybrid system: A method to identify and clone genes for proteins that interact with a protein of interest. Proc. Natl. Acad. Sci. USA.

[22] Fields S., Song O. (1989). A novel genetic system to detect protein-protein interactions. Nature.

[23] Gladue D.P., Baker-Bransetter R., Holinka L.G., Fernandez-Sainz I.J., O’Donnell V., Fletcher P., Lu Z., Borca M.V. (2014). Interaction of CSFV E2 protein with swine host factors as detected by yeast two-hybrid system. PLoS ONE.

[24] Gladue D.P., Holinka L.G., Fernandez-Sainz I.J., Prarat M.V., O’Donnell V., Vepkhvadze N.G., Lu Z., Risatti G.R., Borca M.V. (2011). Interaction between Core protein of classical swine fever virus with cellular IQGAP1 protein appears essential for virulence in swine. Virology.

[25] Zurek N., Sparks L., Voeltz G. (2011). Reticulon short hairpin transmembrane domains are used to shape ER tubules. Traffic.

[26] Grant J.R., Moise A.R., Jefferies W.A. (2007). Identification of a novel immunosubversion mechanism mediated by a virologue of the B-lymphocyte receptor TACI. Clin. Vaccine Immunol. CVI.

[27] Moise A.R., Grant J.R., Vitalis T.Z., Jefferies W.A. (2002). Adenovirus E3-6.7K maintains calcium homeostasis and prevents apoptosis and arachidonic acid release. J. Virol..

[28] Treiman M., Caspersen C., Christensen S.B. (1998). A tool coming of age: Thapsigargin as an inhibitor of sarco-endoplasmic reticulum Ca(^2+^)-ATPases. Trends Pharmacol. Sci..

[29] Burnier M., Centeno G., Burki E., Brunner H.R. (1994). Confocal microscopy to analyze cytosolic and nuclear calcium in cultured vascular cells. Am. J. Physiol..

[30] Homburg S., Visochek L., Moran N., Dantzer F., Priel E., Asculai E., Schwartz D., Rotter V., Dekel N., Cohen-Armon M. (2000). A fast signal-induced activation of Poly(ADP-ribose) polymerase: A novel downstream target of phospholipase c. J. Cell Biol..

[31] Gladue D.P., O’Donnell V., Baker-Branstetter R., Holinka L.G., Pacheco J.M., Fernandez Sainz I., Lu Z., Ambroggio X., Rodriguez L., Borca M.V. (2013). Foot-and-mouth disease virus modulates cellular vimentin for virus survival. J. Virol..

[32] Gladue D.P., O’Donnell V., Baker-Branstetter R., Holinka L.G., Pacheco J.M., Fernandez-Sainz I., Lu Z., Brocchi E., Baxt B., Piccone M.E. (2012). Foot-and-mouth disease virus nonstructural protein 2C interacts with Beclin1, modulating virus replication. J. Virol..

[33] Pinton P., Giorgi C., Siviero R., Zecchini E., Rizzuto R. (2008). Calcium and apoptosis: ER-mitochondria Ca^2+^ transfer in the control of apoptosis. Oncogene.

[34] Giorgi C., Romagnoli A., Pinton P., Rizzuto R. (2008). Ca^2+^ signaling, mitochondria and cell death. Curr. Mol. Med..

[35] Feng P., Park J., Lee B.S., Lee S.H., Bram R.J., Jung J.U. (2002). Kaposi’s sarcoma-associated herpesvirus mitochondrial K7 protein targets a cellular calcium-modulating cyclophilin ligand to modulate intracellular calcium concentration and inhibit apoptosis. J. Virol..

[36] Li J., Huang R., Liao W., Chen Z., Zhang S., Huang R. (2012). Dengue virus utilizes calcium modulating cyclophilin-binding ligand to subvert apoptosis. Biochem. Biophys. Res. Commun..

